# Association of body mass index and outcomes following lobectomy for non-small-cell lung cancer

**DOI:** 10.1186/s12957-018-1394-6

**Published:** 2018-05-11

**Authors:** Cui Wang, Min Guo, Nan Zhang, Gongchao Wang

**Affiliations:** School of Nursing, Shandong University, Jinan, China

**Keywords:** Body mass index, Non-small-cell lung cancer, Outcome, Lobectomy

## Abstract

**Background:**

Obesity and overweight have become increasingly prevalent, but no consensus has been reached regarding the effect of body mass index (BMI) on surgical outcomes. In this study, we sought to examine the influence of BMI on perioperative outcomes in a large cohort of patients with non-small-cell lung cancer (NSCLC) who underwent lobectomy.

**Methods:**

A retrospective study was conducted in 1198 patients who underwent lobectomy for primary NSCLC at Shandong Provincial Hospital between November 2006 and January 2017. BMI was calculated using measured height and weight on admission and categorized as obese (≥ 30 kg/m^2^), overweight (25 to 29.9 kg/m^2^), normal (18.5 to 24.9 kg/m^2^), or underweight (< 18.5 kg/m^2^). Patients’ baseline characteristics and outcomes were abstracted from medical records following institutional review board approval. Endpoints included operative mortality, perioperative complications, and length of stay (LOS). Complications were divided into four groups as respiratory, cardiovascular, other, and overall. Logistic regression models were constructed to assess the association between BMI and adverse outcomes.

**Results:**

When compared with normal BMI, obesity and overweight did not increase the risk of complications in any category, operative mortality, or prolonged LOS. In fact, the incidence of operative mortality and respiratory complications tended to be lower in overweight patients than in normal weight patients (*P* = 0.047 and *P* = 0.041, respectively). Conversely, underweight patients experienced significantly more operative mortality, respiratory complications, and prolonged LOS (*P =* 0.004, *P =* 0.011, and *P =* 0.003, respectively).

**Conclusions:**

Obesity and overweight did not confer adverse surgical outcomes. Underweight patients presented increased risk of respiratory complications, perioperative death, and prolonged LOS. Thus, overweight and obesity should not be a relative contraindication for lobectomy. Meanwhile, nurses and surgeons should focus on perioperative management of underweight patients.

## Background

Lung cancer, as a serious and potentially life-threatening illness, is a major public health problem worldwide. An estimated 1.8 million new cases of lung cancer were diagnosed in 2012 worldwide, accounting for 13% of total cancer diagnoses [1]. In China, lung cancer has the highest death rate among all malignancies, with increasing mortality trends, and is ranked as the leading cause of cancer-related death [2]. Non-small-cell lung cancer (NSCLC) occupies approximately 85% of all lung cancers and has a 5-year survival rate that is generally < 15% [3, 4]. Currently, surgery is the mainstay curative treatment for NSCLC, with lobectomy being one of the most prevalent operative approaches.

Recently, attention has been focused on the factors influencing perioperative outcomes. One factor shown to predict outcomes in lung cancer is body mass index (BMI) [5–8]. However, to date, the data regarding the influence of elevated BMI on surgical outcomes have been mixed. Besides, there are few publications reporting the impact of underweight on surgical outcomes in lung cancer. Obese patients often carry numerous comorbidities, including diabetes and hypertension. Additionally, obesity has long been considered a risk factor for poor outcomes in a variety of surgical procedures, including gynecologic, cardiac, pancreatic, and urologic operations [9]. However, in one previous study of patients with lung cancer who underwent lung resection, Paul et al. disclosed that obesity did not negatively impact perioperative mortality or morbidity [10]. A report specific to lung cancer revealed that increasing BMI was protective against postoperative major morbidity, whereas a very low BMI indicated higher risk [11]. Moreover, prior studies of lung resection suggested that overweight and obesity had favorable surgical outcomes, whereas underweight adversely affected perioperative morbidity and operative mortality [7, 12]. Conversely, in other studies of lung cancer, patients with elevated BMI had increased risk of intrathoracic [5] and respiratory complications [6]. Launer et al. described on patients with lung cancer who underwent lobectomy, indicating that obesity was associated with increased risk of postoperative pulmonary complications, but not mortality or other morbidities after lobectomy [13]. Furthermore, Matsuoka et al. analyzed the outcomes after lung resection at their institution and found that patients with low or high BMI had significantly lower survival relative to patients with normal BMI [14].

Therefore, to better evaluate the effect of BMI in patients with NSCLC undergoing lung resection, we performed this retrospective study to examine the association between BMI and outcomes following lobectomy. We aimed to identify patients who may benefit from perioperative management to achieve improved postoperative outcomes.

## Methods

### Patient selection

A total of 1221 consecutive patients with NSCLC undergoing a first lobectomy via thoracotomy from November 2006 to January 2017 at Shandong Provincial Hospital, affiliated with Shandong University, were selected. These patients underwent lobectomy combined with systematic lymph node dissection. More specifically, the dissected lymph nodes included groups 2, 4, 7, 9, 10, and 11 on the right side and groups 5, 6, 7, 9, 10, and 11 on the left side. We excluded patients whose preoperative BMI was missing (11 patients), who had received chemotherapy and/or radiotherapy prior to surgery (10 patients), and whose tumor stage was stage IV (7 patients). Accordingly, 1198 patients comprised the current study cohort. The project was approved by our Institutional Review Board (Shandong University), which waived the requirement for an individual patient consent because only routine patient data were used for this retrospective analysis.

### Data collection

Baseline demographic, clinical, surgical, and outcome variables were assessed. Demographic characteristics included sex, age, drinking and smoking status, BMI, comorbidities, albumin, forced expiratory volume in 1 s as a percent of predicted (FEV1 predicted), carbon monoxide diffusing capacity as a percent of predicted (DLCO predicted), and American Society of Anesthesiologists (ASA) score. Height and weight were measured on admission, and BMI was calculated as weight in kilograms divided by the square of height in meters. World Health Organization cutoff values were used to categorize BMI as obese (≥ 30 kg/m^2^), overweight (25 to 29.9 kg/m^2^), normal weight (18.5 to 24.9 kg/m^2^), or underweight (< 18.5 kg/m^2^). Comorbidities were classified as diabetes, hypertension, and other (coronary heart disease, heart failure, myocardial infarction, cerebral infarction, cerebral thrombosis, and cerebral hemorrhage). Surgical variables included type of resection, while tumor characteristics included histologic type and tumor stage. Lung cancer was diagnosed by fiber bronchoscopy, pathologic examination, and computed tomography. Tumor stage was established according to the TNM classification of lung cancer proposed by the Union for International Cancer Control, 8th edition.

### Outcomes definition

Our endpoints were operative mortality, perioperative complications, and length of stay (LOS). The primary outcome of interest was operative mortality, which was defined as death occurring within the first hospitalization or within 30 days following the first surgery. Perioperative complications occurred between the end of the first surgery and discharge from the first hospitalization. Complications were divided into groups as follows: cardiovascular (myocardial infarction, shock, pulmonary embolism, heart failure, arrhythmia, cardiac insufficiency, deep venous thrombosis, and cerebrovascular accident), respiratory (acute respiratory distress syndrome, respiratory failure, confirmed or suspected pneumonia, bronchopleural fistula, atelectasis, prolonged air leaks (> 7 days), and hydropneumothorax), other (wound infection, wound dehiscence, pyothorax, chylothorax, fat liquefaction, neurologic complications, pleural effusion, recurrent laryngeal injury, abdominal/urinary tract complications, and miscellaneous), and overall (cardiovascular, respiratory, and others). LOS was calculated as the time from the end of the first surgery until discharge from the first hospitalization. Data regarding patients’ baseline characteristics and outcomes were obtained from medical records.

### Statistical analyses

Statistical analyses were conducted using SPSS version 21.0 (SPSS, Chicago, IL). Continuous normally distributed variables were presented as mean and standard deviation, continuous non-normally distributed variables as median and interquartile range, and categorical variables as number and percentage. Differences in normally and non-normally distributed variables among BMI groups were compared using the analysis of variance and the Kruskal-Wallis test, respectively. Differences in categorical variables among BMI groups were analyzed using the Fisher’s exact test or chi-square test. The Fisher’s exact test was performed when > 20% of the expected value in any cell was < 5. Binary logistic regression was conducted to test the association between adverse outcomes (perioperative complications and operative mortality) and BMI. Unadjusted analyses for adverse outcomes were performed to select variables for inclusion in adjusted analyses. Each selected variable correlated with outcomes having a *P* value of < 0.05. Adjusted odd ratios regarding outcomes were recorded with their exact 95% confidence intervals. The Hosmer-Lemeshow test was performed to test the goodness-of-fit of the models, with larger *P* values considered to have good fit. A two-sided *P* value of < 0.05 was considered statistically significant. To correct for multiple outcome testing, the threshold level for significance was 0.05/3 = 0.017.

## Results

When we stratified BMI into four groups, most patients were normal weight (*n* = 662, 55.3%), followed by overweight (*n* = 428, 35.7%), obesity (*n* = 68, 5.7%), and underweight (*n* = 40, 3.3%) (Table 1). Underweight patients were more likely to have a history of smoking compared with the other BMI groups (*P* = 0.011). Hypertension and diabetes were more common in the obese group relative to the other BMI groups (*P* = 0.000 and *P* = 0.000, respectively). FEV1 predicted, DLCO predicted, and albumin level were lower in underweight group compared with the other BMI groups (*P* = 0.004, *P* = 0.000, and *P* = 0.000, respectively). Right upper lobectomy, advanced cancer stage (stage III), and male sex were more frequent in underweight group than in the other BMI groups (*P* = 0.031, *P* = 0.041, and *P* = 0.020, respectively). Age, drinking status, other comorbidities, ASA score, and histologic type were similar among BMI groups.

**Table 1:** Patient demographics and characteristics

	BMI < 18.5		BMI 18.5–24.9		BMI 25–29.9		BMI ≥ 30		
	Underweight		Normal		Overweight		Obesity		
	*n* = 40 (3.3%)		*n* = 662 (55.3%)		*n* = 428 (35.7%)		*n* = 68 (5.7%)		
Variables	No.	%	No.	%	No.	%	No.	%	*P*
Age, (years), mean (s.d.)	61.3 ± 8.7		58.8 ± 9.5		58.6 ± 9.2		59.6 ± 8.3		0.323
FEV1 predicted, mean (s.d.)	83.3 ± 23.4		90.6 ± 18.6		92.2 ± 16.5		86.6 ± 18.8		*0.004*
DLCO predicted, mean (s.d.)	63.0 ± 14.7		87.2 ± 15.8		83.8 ± 15.5		84.7 ± 15.1		*0.000*
Sex									*0.020*
Male	33	82.5	464	70.1	281	65.7	39	57.4	
Female	7	17.5	198	29.9	147	34.3	29	42.6	
Alcohol-drinking history	26	65.0	339	51.2	197	46.0	31	45.6	0.069
Cigarette-smoking history	27	67.5	413	62.4	233	54.4	33	48.5	*0.011*
Albumin levels (g/dL)									*0.000*
Normal (≥ 3.5)	33	82.5	615	92.9	415	97.0	66	97.1	
Abnormal (< 3.5)	7	17.5	47	7.1	13	3.0	2	2.9	
ASA score									0.231
I—no disturbance	8	20.0	117	17.7	70	16.4	4	5.9	
II—mild disturbance	28	70.0	498	75.2	324	75.7	56	82.4	
III—severe disturbance	4	10.0	47	7.1	34	7.9	8	11.8	
Hypertension	2	5.0	112	16.9	115	26.9	36	52.9	*0.000*
Diabetes mellitus	0	0.0	51	7.7	57	13.3	12	17.6	*0.000*
Other comorbidities	2	5.0	28	4.2	19	4.4	3	4.4	0.956
Type of resection									*0.031*
Left upper lobectomy	8	20.0	118	17.8	80	18.7	15	22.1	
Left lower lobectomy	9	22.5	157	31.7	96	22.5	19	27.9	
Right upper lobectomy	15	37.5	160	24.2	135	31.5	22	32.4	
Right middle lobectomy	0	0.0	60	9.1	24	5.6	5	7.4	
Right lower lobectomy	8	20.0	167	25.2	93	21.7	7	10.3	
Histologic type									0.320
Adenocarcinoma	22	55.0	387	58.5	269	62.9	47	69.1	
Squamous cell carcinoma	18	45.0	263	39.7	150	35.0	21	30.9	
Other type	0	0.0	12	1.8	9	2.1	0	0.0	
Tumor stage									*0.041*
I	14	35.0	294	44.4	217	50.7	35	51.5	
II	12	30.0	213	32.2	101	23.6	17	25.0	
III	12	35.0	155	23.4	110	25.7	16	23.5	

Italic values: *P* < 0.05 was significant. Categorical variables were compared using chi-square test. Continuous variables were compared using analysis of variance

*ASA score* American Society of Anesthesiologists score, *BMI* body mass index, *DLCO predicted* carbon monoxide diffusing capacity as a percent of predicted, *FEV1 predicted* forced expiratory volume in 1 s as a percent of predicted, *s.d* standard deviation

The details concerning unadjusted outcomes are summarized in Table 2. The incidence of overall complications was 23.0% (*n* = 276). Other complications occurred in 11.9% (*n* = 143) of patients, followed by respiratory (7.3%, *n* = 87) and cardiovascular complications (7.0%, *n* = 84). The mortality rate of our patient population was 1.8% (*n* = 22). The proportion of patients experiencing operative mortality or respiratory complications decreased across increasing BMI groups (*P* = 0.002 and *P* = 0.001, respectively). Underweight patients had the longest LOS, with a median of 10 days, compared with 9 days among the other BMI groups (*P* = 0.003). There were no significant differences in overall, cardiovascular, or other complications among BMI groups.

**Table 2:** Unadjusted postoperative outcomes stratified by weight group

	BMI < 18.5		BMI 18.5–24.9		BMI 25–29.9		BMI ≥ 30		
	Underweight		Normal		Overweight		Obesity		
	*n* = 40 (3.3%)		*n* = 662 (55.3%)		*n* = 428 (35.7%)		*n* = 68 (5.7%)		
Outcomes	No.	%	No.	%	No.	%	No.	%	*P*
LOS (day), median (IQR)	10 (9–12)		9 (8–11)		9 (8–11)		9 (8–10)		*0.003*
Operative mortality	4	10.0	15	2.3	2	0.5	1	1.5	*0.002*
Perioperative complications									
Cardiovascular	5	12.5	47	7.1	25	5.8	7	10.3	0.635
Respiratory	9	22.5	54	8.2	20	4.7	4	5.9	*0.001*
Other	3	7.5	77	11.6	51	11.9	12	17.6	0.405
Overall	12	30.0	149	22.5	93	21.7	22	32.4	0.176

Italic values: *P* < 0.05 was significant. Continuous variable were compared using Kruskal-Wallis H test. Categorical variables were compared using chi-square test or Fisher’s exact test

*BMI* body mass index, *LOS* length of stay, *IQR* interquartile range

Adjusted outcomes are listed in Table 3. When compared with the normal BMI group, respiratory complications and operative mortality were significantly lower in the overweight group (*P* = 0.041 and *P* = 0.047, respectively). Adjusted odds of operative mortality or respiratory complications tended to be higher in underweight group (*P* = 0.004 and *P* = 0.011, respectively). Overall, cardiovascular and other complications were comparable despite BMI.

**Table 3:** Adjusted multivariable regression analysis of postoperative outcomes according to BMI category

	Operative mortality		Respiratory complications		Cardiovascular complications		Other complications		Overall complications	
BMI category	OR	95% CI	OR	95% CI	OR	95% CI	OR	95% CI	OR	95% CI
Normal	Reference		Reference		Reference		Reference		Reference	
Underweight	4.39*	*1.31–14.72*	*2.88* ^*#*^	*1.27–6.50*	1.70	0.61–4.73	0.55	0.16–1.83	1.31	0.64–2.67
Overweight	0.22**	*0.05–0.98*	*0.57* ^*##*^	*0.34–0.98*	0.87	0.52–1.45	1.05	0.71–1.53	0.98	0.73–1.32
Obesity	0.70	0.09–5.51	0.71	0.25–2.05	1.56	0.67–3.79	1.60	0.82–3.15	1.68	0.97–2.92

Italic values: *P* < 0.05 was significant. Adjusted for age, sex, alcohol-drinking history, cigarette-smoking history, other comorbidities, tumor stage, and FEV1 predicted

*BMI* body mass index, *CI* confidence interval, *OR* odd ratio

^#^*P* = 0.011; ^##^*P* = 0.041; *P = 0.004; **P = 0.047

Single complications are disclosed in Table 4. Single complications were ranked by decreasing frequency when > 5 events occurred in the normal BMI group. Pneumonia and prolonged air leaks developed more frequently in underweight group when compared with other BMI groups (*P* = 0.001 and *P* = 0.029, respectively). When performing multiple comparisons, a significant protective effect of overweight was observed for prolonged air leaks as compared with normal BMI (*P* = 0.013). Furthermore, the incidence of pneumonia tended to be higher in underweight group compared with normal weight group (*P* = 0.010).

**Table 4:** Unadjusted single complications stratified by weight group

	BMI < 18.5		BMI 18.5–24.9		BMI 25–29.9		BMI ≥ 30		
	Underweight		Normal		Overweight		Obesity		
	*n* = 40 (3.3%)		*n* = 662 (55.3%)		*n* = 428 (35.7%)		*n* = 68 (5.7%)		
Single complications	No.	%	No.	%	No.	%	No.	%	*P*
Pleural effusion	2	5.0	50	7.6	28	6.5	9	13.2	0.243
Arrhythmia	3	7.5	35	5.3	18	4.2	5	7.4	0.735
Pneumonia	6*	15.0	28	4.2	14	3.3	2	2.9	*0.029*
Prolonged air leaks	40	10.0	19	2.9	3**	1.5	1	2.3	*0.001*
Cardiac insufficiency	1	2.5	11	1.7	5	1.2	1	1.5	0.496
Heart failure	0	0.0	7	1.1	0	0.0	0	0.0	0.076
Chylothorax	10	2.5	7	1.1	6	1.4	4	5.9	0.062
Atelectasis	00	0.0	7	1.1	6	1.4	2	2.9	0.481

Italic values: *P* < 0.05 was significant. Difference statistically significant (reference group: normal). To correct for multiple outcome testing, the threshold for significance level was 0.05/3 = 0.017. *P = 0.010, **P = 0.013. Categorical variables were compared using chi-square test or Fisher’s exact test

*BMI* body mass index

## Discussion

The prevalence of obesity and overweight are showing an upward trend. The present study observed that patients with elevated BMI accounted for 41.1% of our subjects. Thoracic surgeons will encounter more obese and overweight patients with NSCLC in the future. Therefore, it is important to fully understand the effect of BMI on perioperative outcomes in such patients. We carried out this retrospective analysis using our institutional database to further explore the association between BMI and perioperative outcomes. It was interesting to find that overweight and obesity did not have any negative impact on outcomes after lobectomy. In fact, a significant protective effect of overweight was observed for respiratory complications and operative mortality. Meanwhile, underweight patients presented increased risk of respiratory complications, operative mortality, and prolonged LOS.

Notably, despite overweight and obese patients having more comorbidities, including hypertension and diabetes, they did not have a worse prognosis compared with normal weight patients. This result correlated with findings obtained from Mungo et al. [8], who investigated the National Surgical Quality Improvement Program database and confirmed that obesity did not confer a greater risk of mortality or morbidity after lung resection in unadjusted and adjusted analyses. Similarly, a retrospective study that divided surgical patients with lung cancer into two BMI groups (≥ 30 and < 30 kg/m^2^) observed that even though patients with BMI ≥ 30 kg/m^2^ were more likely to have hypertension, diabetes, and renal impairment, they demonstrated a better survival rate before and after propensity matching with a cohort of BMI < 30 kg/m^2^ [15]. A systematic review concluded that obesity had favorable effects on in-hospital outcomes and long-term survival in surgical patients with lung cancer [4]. Sepesi et al. found that higher BMI correlated with improved long-term overall survival after surgical resection for NSCLC [16]. Ferguson et al. observed that patients who were overweight or mildly obese were at somewhat lower risk for complications following lung resection compared with patients in other BMI groups [7]. Dhakal et al. suggested no significant differences in perioperative mortality, morbidity, or LOS in patients with BMI ≥ 25 kg/m^2^ compared with patients with BMI < 25 kg/m^2^ [9].

A potential mechanism may explain the complex interaction between elevated BMI and perioperative outcomes. First, because BMI cannot discriminate between fat mass and lean mass, it might be that overweight and obese patients did not have more fat, but instead, had preserved or increased lean body mass, while lean mass correlated with a protective effect [17]. Research has found that such “obesity paradox” is present in patients with cancer when obesity is defined by BMI [18]. Li et al. conducted a systematic review, suggesting that obesity could be a strong predictor of favorable long-term prognosis in patients who underwent lung cancer surgery, and noted that the obesity paradox did have the potential to exist in patients with lung cancer [4]. The paradoxical benefit of obesity has also been found in a wide range of cardiovascular and surgical populations [4]. Second, patients with elevated BMI tended to have an earlier cancer stage, relatively low energy expenditure due to the earlier cancer stage, and higher preoperative albumin level, indicating that such patients may have better nutrition and tolerance of surgery. Furthermore, patients with elevated BMI tend to have greater adipose tissue, making them less likely to suffer from energy deficit and more likely to have a sufficient nutritional reserve for further postoperative treatment [15]. Moreover, although weight loss caused by cancer is a risk factor for poor prognosis [4, 15], patients with high BMI may not experience weight loss prior to surgery. Additionally, the inflammatory status of obesity is low grade and chronic, which could induce adiposity to secrete adipokines and cytokines, resulting in a more appropriate inflammatory response to surgical trauma [19]. Additionally, adipocytes are known to regulate inflammation and endovascular homeostasis and to increase insulin sensitivity. Lipoproteins, which are often increased in obesity, can bind to and neutralize circulating endotoxins. Thus, the protective effect of obesity and overweight may be attributable to genetics and unknown factors [17]. Finally, physicians often pay more attention to patients with high BMI and often advise them to perform regular exercise and cardiac rehabilitation and to form healthy dietary habits. On the other hand, few patients with normal BMI receive such attentive care and health tips, so they tend to ignore their own health until they suffer from severe disease [4]. We found a significant protective effect of overweight for operative death and respiratory complications. However, our findings do not provide insight concerning the protective effect in obese group compared with the normal BMI group. It may be explained by the low ratio of obese patients in our study and/or the low rate of postoperative complications after lobectomy in general.

In contrast, another previous study showed that risk of respiratory complications in patients with BMI ≥ 25 kg/m^2^ undergoing pneumonectomy for lung cancer was 5.3 times higher than that in patients with BMI < 25 kg/m^2^ [6]. However, their study focused on pneumonectomy, which is different from lobectomy. Additionally, only 154 patients with NSCLC comprised their study cohort.

Our data found that being underweight was associated with increased risk of mortality, morbidity, and prolonged LOS. The outcomes of our study are similar to the studies by Ferguson et al. [7] and Mungo et al. [8]. Additionally, Nakagawa et al. reported on outcomes after lung resection, concluding that the underweight group had a significantly worse prognosis than the other BMI groups for disease-free survival in univariate and adjusted analyses [20]. There are various reasons for this. First, underweight patients had a more advanced cancer stage (stage III). Because advanced cancer requires higher basal metabolic demand and energy consumption [21, 22], these patients conferred higher risk of preoperative rapid weight loss, affecting their health and immune system and leaving them in an adverse physiologic state [8, 15]. Hence, underweight patients may experience weight loss caused by cancer, leading to adverse outcomes. The prior study revealed that weight loss was considered to be a risk factor for poor prognosis [4]. Moreover, the present study found that underweight patients were more likely to be former smokers with worse pulmonary function, which may explain the higher rate of respiratory complications in the low BMI group. Thomas et al. suggested that interventions such as smoking session programs and pulmonary functional exercise should be provided for underweight patients, even though no firm conclusions can be reached regarding their effectiveness based on current evidence [12]. Furthermore, our data showed a high proportion of albumin deficiency in underweight patients, and low albumin is known to have a deleterious impact on mortality and morbidity in many conditions [23, 24]. Additionally, nutritional depletion is generally accepted as a predictor of poor prognosis in surgical patients [4]. Nutritional deficiency is relevant to weakness of expiratory muscles and impaired immune response, which may work together to justify the positive relationship between low BMI and adverse outcomes [25]. Research on nutrition interventions for patients with lung cancer has explored supplementation with fish oil, intensive dietary counseling, and interdisciplinary models of nutrition and has shown improved outcomes with these interventions [26].

Potential limitations of our study include its retrospective design and possible selection bias. Furthermore, all study subjects were admitted to the same institute, which may affect the objectivity of the results. Moreover, there were few obese and underweight patients in our study; thus, our results need to be confirmed in a larger-scale study. In addition, although peripheral fat has a protective effect, BMI cannot distinguish between visceral and peripheral adiposity [7]. Therefore, other measures, including waist-to-hip ratio and waist-to-height ratio, should be assessed in subsequent studies; computed tomography can also provide accurate measurement of intra-abdominal fat content [18]. Moreover, low BMI is not always a surrogate marker of impaired nutrition. Consequently, future studies need to perform a more complete assessment regarding patients’ nutritional status and to distinguish between protein and caloric malnutrition. Other laboratory values that should be taken into account are serum transferrin, glucose, electrolytes, blood urea nitrogen, creatinine, iron, vitamin levels, calcium, magnesium, and others. Future studies could perform an analysis regarding the association between a specific complication and a specific type of malnutrition or deficiency of a specific nutrient. Additionally, underweight patients had adverse prognosis as a result of preoperative weight loss caused by cancer. However, weight fluctuation was not taken into consideration, as data regarding preoperative weight change were unavailable. Preoperative weight fluctuation needs to be assessed in the future. Finally, our results cannot be extrapolated to form conclusions on long-term outcomes; thus, the association between BMI and long-term outcomes awaits to be further elucidated. The advantages of our study include the large and homogeneous patient cohort, with exclusion of patients undergoing induction therapy before surgery, as chemotherapy and/or radiotherapy exert a profound effect on nutritional status and BMI.

## Conclusions

To conclude, despite the above limitations, we observed that overweight and obesity did not negatively affect perioperative outcomes. However, being underweight was associated with adverse surgical outcomes, including respiratory complications, operative death, and prolonged LOS. Such information may help thoracic surgeons to facilitate appropriate treatment for patients receiving lung resection. Overweight and obesity should not be a prohibitive risk factor for lung cancer surgery. Meanwhile, greater attention should be paid to perioperative management of underweight patients. Ultimately, further well-designed, prospective, large-scale studies are needed to validate the effect of BMI on surgical outcomes in lung cancer.

## Abbreviations

BMI: Body mass index
DLCO predicted: Carbon monoxide diffusing capacity as a percent of predicted
FEV1 predicted: Forced expiratory volume in 1 s as a percent of predicted
LOS: Length of stay
NSCLC: Non-small-cell lung cancer
